# In Vivo Insights into the Role of Astragaloside IV in Preventing and Treating Civilization Diseases: A Comprehensive Review

**DOI:** 10.3390/ijms26094250

**Published:** 2025-04-29

**Authors:** Katarzyna Stępnik, Agata Jarząb, Rafał Niedźwiadek, Anna Głowniak-Lipa, Kazimierz Głowniak, Wirginia Kukula-Koch

**Affiliations:** 1 Department of Physical Chemistry, Institute of Chemical Sciences, Faculty of Chemistry, Maria Curie-Sklodowska University in Lublin, Pl. M. Curie-Skłodowskiej 3, 20-031 Lublin, Poland; 2 Department of Biochemistry and Molecular Biology, Medical University of Lublin, Chodźki 1, 20-093 Lublin, Poland; 3 Department of Cosmetology, University of Information, Sucharskiego 2, 35-225 Rzeszów, Poland; 4 Department of Pharmacognosy with Medicinal Plants Garden, Medical University of Lublin, Chodźki 1, 20-093 Lublin, Poland; virginia.kukula@gmail.com

**Keywords:** *Astragalus* spp., astragaloside IV, anticancer properties, anti-inflammatory action, natural products, cognition enhancing properties, saponins

## Abstract

Civilization diseases are a growing and global health problem in modern societies. Neurological disorders, cancer, and inflammatory diseases affect a large group of patients around the world. Therefore, it is of utmost importance to search for novel drugs, lifestyle tips, and foods that can help restore balance in the living organism, promote the efficiency of the immune system, and provide satisfactory prophylactic measures. Astragaloside IV (ASIV)—a triterpenoid saponin from *Astragalus* species, one of the world’s most widely used herbs—has been shown to have a variety of biological properties, including anti-inflammatory, antioxidant, antitumor, and neuroprotective effects. In recent years, the number of in vivo studies on this active ingredient in the scientific literature has increased considerably. The aim of this review was therefore to compile the existing knowledge on the use of this compound in the treatment of selected diseases of civilization—cancer, neurological disorders, and inflammatory diseases—in vivo.

## 1. Introduction

Astragaloside IV, a triterpenoid saponin from *Astragalus* species, shows multiple anticancer and therapeutic effects targeting inflammatory and tumor-related signaling pathways. Saponins are naturally occurring metabolic products that are widespread in plants. They are mainly found in roots, stems, and leaves. Herbal medicinal plants that contain triterpenoid saponins include *Glycyrrhizaeradix*, *Saponariaeradix*, *Primuleaeradix*, *Herniariaeradix*, and *Hedereafolium* [1]. Saponins are structurally composed of two parts: a sapogenone (an aglycone part) and a saccharide part (a glycone part). Due to their amphiphilic structure, they have a high ability to reduce surface tension and form a stable and dense foam [1,2,3,4,5].

This group of compounds is characterized by very different physicochemical properties, such as a bitter and sweet taste [1,6,7,8] or the ability to form foam and emulsion [1,9]. In addition, numerous studies have demonstrated their pharmacological [1,10], insecticidal, and, in some cases, hemolytic properties [1,11,12]. Due to their amphiphilic nature, they can be dissolved in water to form acidic solutions and also in organic solvents [1].

**Figure 1:**
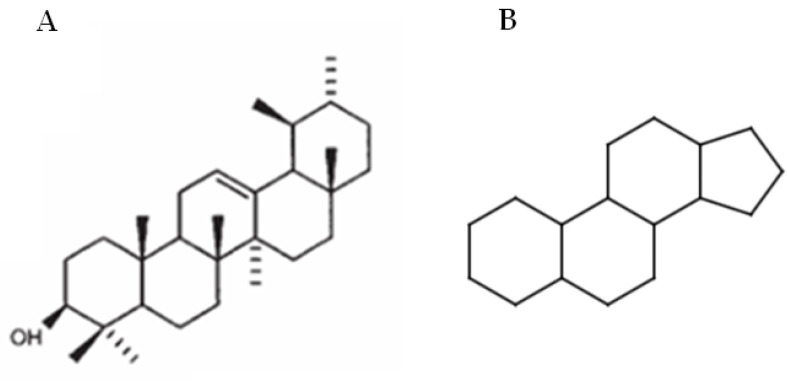
Chemical structures of α-amyrin (**A**) and sterane core (**B**).

Looking at the chemical structure of the saponins found in plants, two main types can be distinguished: steroidal and triterpenoid saponins. This subdivision refers to the type of aglycones, which are characterized by a different number of rings that form the aglycone part. Both groups areoxydosqualene derivatives, which contain thirty carbon atoms in the molecule [1,13]. In the triterpenoid saponins, the ring is usually formed by α-amyrin with 30 carbon atoms, whereas steroidal saponins typically contain a 17-carbon sterane core to which a 10-carbon side chain is attached at the C17 position (Figure 1). In some cases, this side chain forms a cyclic structure (e.g., in spirostanol saponins). The two groups also differ in the type and number of sugars attached to the aglycone moiety, in the number and position of double bonds, and in the number and position of functional groups in the molecule [1].

Based on the number of saccharide chains present in the saponin molecule, a distinctioncan be made between monodesmosides (with one sugar chain), bidesmosides (with two attached sugar groups), and tridesmosides (with three sugar units). The most frequently occurring saccharides in the glycone part are: L-fucose, L-arabinose, L-rhamnose, D-glucose, D-xylose, D-galactose, D-galacturonic acid, and glucuronic acid. The sugar and non-sugar parts are linked by an ether bond and more rarely by an ester bond, whereby the hydroxyl groups of the saccharide occur in the enylated form [1,14]. Saponins can form bonds with sterols, which also include cholesterol, a component of the biological membranes of living organisms. These special properties of saponins, such as the interaction with membranes or the binding to sterols, lead to an increase in the permeability of these substances to the cell space as a result of partial membrane damage [1].

Studies demonstrate the efficacy of astragaloside IV in modulating macrophage polarization, reducing inflammation, and restoring homeostasis in conditions such as inflammatory bowel disease (IBD) through mechanisms related to STAT signaling, among others. ASIV also shows anti-tumor activity in gastric, colon, and liver cancer by affecting key molecular signaling pathways such as miRNA regulation, mitochondrial apoptosis, and immune checkpoint suppression. It promotes apoptosis, autophagy, and immune response while suppressing tumor growth and immune evasion. ASIV also mitigates chemotherapy-induced damage to bone marrow aspirates and promotes hematopoiesis.

As the following summary shows, ASIV reduces the proliferation, migration, and viability of various cancer cells, including breast, lung, colon, cervical, and liver cancers, often by inducing apoptosis and modulating key signaling pathways such as PI3K/AKT/mTOR, TGF-β1/FAK/AKT and AMPK. ASIV also enhances the effect of chemotherapeutic agents such as cisplatin and gefitinib by overcoming drug resistance through autophagy, ER stress, and SIRT6 activation. It also inhibits metastasis by suppressing M2 macrophage polarization and reducing markers of angiogenesis.

These results position ASIV as a promising therapeutic for cancer and inflammatory diseases, as demonstrated by the key new scientific findings.

To compile the latest and most relevant publications on the biological properties of astragaloside IV and its application in the treatment of civilization diseases, a comprehensive study was conducted using specialized scientific databases such as Web of Science, Scopus, and PubMed. To obtain the desired keywords, the search was conducted using specific terminology that was consistent with MeSH. The databases were searched for the following terms, both individually and in context: ‘astragalus’, ‘astragaloside IV’, ‘diseases of civilization’, ‘Alzheimer’s’, ‘cognitive impairment’, ‘neurology’, ‘cancer’, ‘chemoprevention’, ‘bioavailability’, ‘inflammation’, ‘anti-inflammatory’, ‘free radicals’, ‘physico-chemical properties’, ‘Fabaceae’, ‘Astragalus membranaceus’, ‘neoplasms’, ‘prevention’, ‘antineoplastic agents’, ‘pharmacology’, and ‘natural product therapy’. The keyword ‘in vivo’ was always included in the search, as the main idea in preparing this review manuscript was to summarize the most important findings from in vivo studies. The exclusion criteria included studies on mixtures such as plant extracts or other mixtures without clear evidence on the properties of astragaloside IV itself and articles without peer review (comments, letters to the editor).

The data on the most representative mechanisms of action of astragaloside IV and the scientific models used in the in vivo animal studies are presented in the tables and figures.

## 2. Physicochemical Properties

The European Pharmacopoeia contains the monograph of the whole dried root of Astragalus mongholicus, which should be standardized to the content of astragaloside IV, which should not be less than 0.04% [15].

In traditional Chinese medicine, Astragalus was used in the form of a dry root, as a powder, or as a decoction. It was prescribed alone or in combination with other herbs, most commonly with Angelica sinensis, Panax ginseng, or Ligusticum lucidum [16]. In addition to flavonoids (such as kaempferol, quercetin, isorhamnetin, or calicosin), biogenic amines (asparagine, glutamic acid, canavanine, proline, arginine, γ-aminobutyric acid), sugars, andisoflavones (formononetin, kumatakenin), the plant is a rich source of triterpenoid saponins, namely the astragalosides I–VIII (ASI-ASVIII) and their esters, the agroastragalosides I-VI, and the astramembranins I and II, which largely determine the quality of the root as a medicinal raw material, with astragaloside IV being the most important component of this complex [16,17,18].

**Figure 2:**
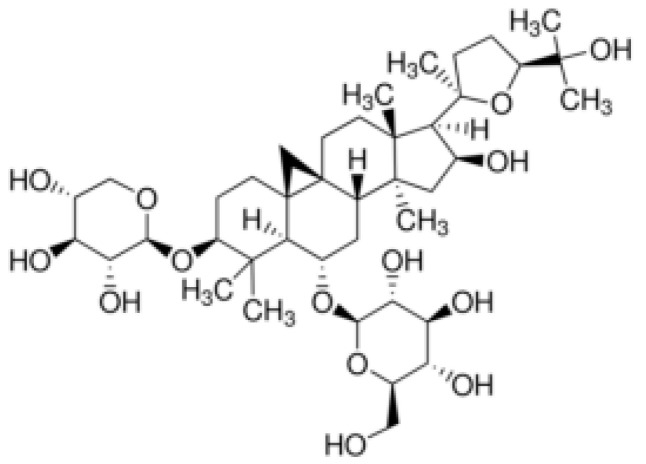
Chemical structure of astragaloside IV.

Astragaloside IV (3-O-β-D-xylpyranosyl-6-O-β-D-glucopyranosyl-cycloastragenol) is a tetracyclic triterpenoid saponin with the character of a lanolin alcohol found in Astragalus species. It is a white to light yellow solid with good solubility in methanol, acetone, and ethanol. It is practically insoluble in organic solvents with low polarity, such as chloroform and ethyl acetate [19,20]. The limitations of water solubility and thermal stability of astragaloside IV can be overcome by extraction and separation methods, which include high-speed centrifugation, water or ultrasonic extraction, ultrafiltration, alcohol precipitation, or refluxing [19,21]. The structure of ASIV is shown in Figure 2.

The active form of astragaloside IV is cycloastragenol, which is capable of activating telomerase, lengthening telomeres, and exhibiting immunomodulatory, antiviral, anti-fat accumulation, anti-ischemia and hypoxia, antioxidant, anti-apoptotic, neuroprotective, cardioprotective, hepatoprotective, and anticancer properties [19,20,22,23]. A summary of the most important physicochemical properties including the steric, lipophilic, and electronic propertiesof ASIV can be found in Table 1.

## 3. Bioavailability

A study on the bioavailability of astragaloside IV by Yuan et al. showed that the oral bioavailability in beagle dogs was only 7.4%, while a value of 3.66% was determined in rats [19,26]. The low absorption and bioavailability of this biologically active substance is most likely due to its high molecular weight, poor intestinal permeability, paracellular transport into organisms, and low ability to cross biological membranes [19,27].

ASIV was administered orally to beagle dogs at a dose of 10 mg/kg. As a result of the experiment, the elimination half-life was found to be 229.71 min and the area under the curve of time dependence of astragaloside concentration was 204.05 μgh/mL. When this compound was administered intravenously at doses of 0.5, 1, and 2 mg/kg, the T1/2 values were 177.18, 196.58, and 241.59 min, respectively, and the AUC was 126.24, 276.28, and 724.51 μgh/mL, respectively [19,26].

After the intravenous administration of ASIV at a dose of 4 mg/kg to rats, the highest accumulation of the chemical was observed in the kidneys, spleen, liver, heart, and lungs. The mean concentrations of astragaloside IV in these organs together with the standard deviations are shown in Table 2.

The degree of binding of ASIV to plasma proteins in rats in the concentration range of 250–1000 ng/mL is about 90% [19,26]. About 50% of the astragaloside administered is metabolized in the body, mainly removed by the liver, and excreted via the bile, urine, and feces. Daily excretion via these routes amounted to 31.92%, 13.43%, and 31.41% in male rats and 36.20%, 21.77%, and 31.84% in female rats [19,28].

No side effects or adverse reactions were observed after the intravenous administration of ASIV to dogs and rats over a period of 3 months. Adverse effects such as nephrotoxicity and hepatotoxicity were observed in rodents after the oral administration of a dose of 10 mg/kg per day over a period of 14 weeks [19,29,30].

A study was also conducted to determine the average maximum concentration of ASIV in human plasma in healthy patients by administering astragaloside IV extract intravenously in doses of 200, 300, 400, and 500 mL. The results of these studies are shown in Table 3.

The average cumulative urinary excretion of ASIV in humans is about 4% per day after a dose of 500 mL. After the administration of variable doses in the range of 200–600 mL or the repeated administration of a fixed dose over a period of 7 days, ASIV does not accumulate in human plasma [19,31], demonstrating the safety of astragaloside IV as a drug.

Zang et al. conducted a study on the usefulness of high-performance liquid chromatography coupled with tandem mass spectrometry in the quantification of astragaloside IV. In the concentration range of 1–500 ng/mL, the range of the calibration curve was linear, the detection limit for astragaloside was set at 0.5 ng/mL, and the recovery rate was 92.5%. No interference was observed in the analyses. In vivo studies in beagle dogs and rats showed that the excretion of the drug follows the biphasic AUC curve [29,30]. The results obtained are shown in Table 4.

In the human studies conducted by liquid chromatography–electrospray ionization–mass spectrometry (LC-ESI-MS), EBA values, the degree of binding to ASIV proteins in the dose range of 200–1000 ng/mL, AUC, Cmax, Tmax, and T1/2 were found to be 7.4%, 90%, 134.73 ng/mL, 1.5 h, and 5.45 h, respectively. It was also found that the transport of ASIV occurs in a passive manner [26,27,29,33]. Astragaloside IV distributes most extensively in the lungs, liver, and kidneys, whereas it distributes only to a very small extent in the brain, demonstrating its low permeability of the blood–brain barrier [28,29,34].

The way in which this biologically active compound is excreted from the body has also been investigated. ASIV is removed from the body relatively quickly, as shown by elimination half-lives in rats and dogs of 34–131.6 and 50.2–68.8 min, respectively. After one day, a recovery rate of 30.8 was found in bile, while only 50% of the parent form of astragaloside IV was detected in urine and feces. This means that half of the ingested dose of this saponin is metabolized at the cellular level [29,34,35]. It is metabolized via intestinal bacteria and hepatobiliary circulation by deglycosylation into five main metabolites. Antibiotics have a significant effect on inhibiting the uptake of the parent form of ASIV by the intestinal bacterial flora [29,36,37].

Qing’s team conducted a study to search for a better-absorbed form of ASIV that exhibits a broad spectrum of pharmacological effects. Experimentally, the researchers succeeded in converting ASIV to its carboxylic acid derivative called astragalosidic acid through the oxidative 2,2,6,6-tetramethylpiperidine 1-oxyl radical (TEMPO)-mediated conversion. In contrast to the original form of this saponin, this ASIV derivative is readily soluble in water and thus soluble in body fluids [29,38]. The quantities characterizing the pharmacokinetics of this derivative have been determined. The average maximum concentration reached approximately one hour after oral administration was 248.7 ng/mL, and the half-life was in the range of 1.55–4.49 h. A double absorption peak was observed on the ASIV plasma concentration versus time curve after oral administration. The EBA of astragalosidic acid is twice as high as the EBA of astragaloside IV, and it is also better absorbed by the intestine, making it a potential drug candidate. Studies in rats have shown that no adverse side effects were observed even at a dose of 5000 mg/kg, confirming the safety of astragalosidic acid in pharmaceutical preparations [29,39,40].

A study by Gui et al. found no hepatotoxicity or nephrotoxicity [24,41] of ASIV in young and adult animals at safe doses equivalent to 35 times and 70 times the safe dose in humans (570 mg/kg), respectively [24,42]. However, the toxicity of astragaloside IV in pregnant animals was demonstrated at an oral dose of 1 mg/kg per day. Such treatment led to delays in the coat appearance and neurological development of the young animals. Therefore, caution should be exercised when treating pregnant animals with this substance [24,43,44]. Zhu et al. observed similar adverse effects with the intravenous administration of this saponin, but no teratogenicity of ASIV was observed [24,45].

In our previous studies, we dealt with the determination of lipophilicity using biomimetic HPLC systems. A cholesterol-bound stationary phase and an IAM column were used. These types of stationary phases have pseudo membrane properties so that even newly synthesized compounds can be investigated with them [46,47,48,49,50,51]. Based on the Soczewinski–Wachtmeister equation, the value of logkw was determined, which is considered an alternative to logPow, a descriptor of lipophilicity [46,52]. The value of this parameter for the IAM column is 1.727, while for the CHOL column, it is 2.668 [46].

## 4. Anti-Inflammatory Potential

**Figure 3:**
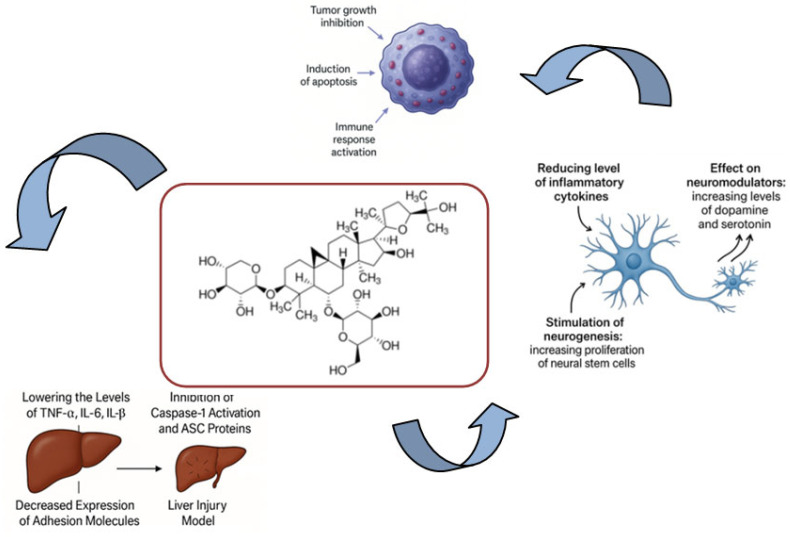
Selected mechanisms of action of astragaloside IV in the treatment of civilization diseases.

Various anti-inflammatory mechanisms have been elucidated for astragaloside IV based on the scientific literature (see Figure 3). The majority of these findings stem from extensive in vitro studies. However, approximately 40 entries were identified that directly or indirectly describe the inflammation-reducing effects of ASIV in the treatment of various ailments in vivo.

Wang and colleagues [53] demonstrated a significant effect of ASIV on renal tubular damage in diabetic kidney disease. Their studies involving rats fed a high-fat diet and administered ASIV at two doses (10 and 20 mg/kg body weight/day) for eight weeks revealed that the saponin reduced blood glucose levels, ameliorated lipid metabolism disturbances, and decreased mitochondrial-derived reactive oxygen species (ROS). Additionally, ASIV reduced the NLRP3-mediated inflammatory cascade associated with diabetic kidney disease. Further investigations conducted by the authors in an in vitro model demonstrated ASIV’s ability to interact with the FATP2-mediated transport of fatty acids by inhibiting its activity. Similar conclusions were drawn from the study by Zha and coworkers [54]. The administration of ASIV in the acute kidney injury model indicated the anti-inflammatory properties of the saponin. This included the reduction inIL-18, IL-1beta, GSDMD-N, and cleaved caspase-1 levels in SpragueDawley rats treated with ASIV. Renal dysfunction parameters such as SCR and BUN were also decreased compared to the non-treated group, suggesting that the saponin in question is an efficient candidate to inhibit pyroptosis.

The anti-inflammatory properties of ASIV were highlighted as beneficial for modulating cisplatin-induced liver injury in the study by Guo et al. [55]. The authors observed a significant improvement in inflammatory and oxidative stress conditions in mice treated with 40 or 80 mg/kg b.w. ASIV. Based on these results, astragaloside IV proved to be an efficient ferroptosis inhibitor, also thanks to its ability to block the PPARalpha/FSP1 signaling pathway.

Current research indicates that monocytes infiltrating the tissue undergo differentiation into macrophages that exhibit different characteristics corresponding to either the M1 or M2 type. The accumulation of M2-like pro-resolving macrophages in the intestinal environment appears to play a crucial role in restoring balance and homeostasis in intestinal tissue. The therapeutic effect and underlying mechanism of ASIV in experimental colitis have been investigated, clearly demonstrating the efficacy of ASIV in the treatment of inflammatory bowel disease (IBD). It was observed that administration of ASIV resulted in a shift of macrophages from a pro-inflammatory M1 state (MLPS) to a pro-resolving M2 state (MIL-4). Flow cytometry results also showed that ASIV treatment reduced the number of bone marrow-derived pro-inflammatory macrophages while promoting their functional maturation. The activation of certain transcription factors within this macrophage-specific gene expression framework likely drives macrophage polarization. These include factors such as the STAT family, the nuclear receptor PPARγ, the CREB–C/EBP axis, and various interferon-regulatory elements. This showed a significant increase in p-STAT1 levels in the DSS group, which was reversed by ASIV treatment. In contrast, p-STAT3 levels increased after ASIV treatment. STAT3 is an important immunoregulatory transcription factor that plays a crucial role in IBD. It is therefore hypothesized that ASIV treatment may help to balance the activity of p-STAT1 and p-STAT3. Mechanistic studies revealed that ASIV binds specifically to STAT1 as a ligand, promotes the dephosphorylation of Tyr701, and leads to the inactivation of STAT1. It was also confirmed that the regulatory effect of ASIV on macrophage phenotype was abolished when STAT1 signaling was inhibited in a rescue experiment. Taken together, this suggests that ASIV can influence the macrophage phenotype via the modulation of the STAT1 signaling pathway [56].

The anti-inflammatory potential of ASIV was assessed in ApoE-deficient (ApoE−/−) and C57BL/6J murine models treated with BuyangHuanwu Decoction or three glycosides, which constitute the primary active components of this formulation: ASIV, paeoniflorin, and amygdalin [57]. Results obtained from the atherosclerotic inflammation model substantiated the efficacy of both the decoction and the individual glycosides in mitigating the inflammatory response associated with hyperlipidemia and the development of atheromatous plaques. All glycosides and the decoction exerted significant effects on the JAK/STAT signaling pathway, evidenced by decreased expression levels of JAK2, STAT1, STAT3, vascular cell adhesion molecule-1 (VCAM-1), and intercellular adhesion molecule-1 (ICAM-1) as well as reduced concentrations of interleukin-6 (IL-6) and tumor necrosis factor-alpha (TNF-α) in the aortic wall. Notably, analogous outcomes were observed for the glycosides and the decoction concerning blood lipid profiles, inflammatory pathways, and adhesion molecules, indicating an anti-inflammatory and anti-atherosclerotic effect of ASIV and underscoring its pivotal role in the regulation of cardiovascular health.

Furthermore, the mechanism of action attributed to astragaloside IV includes its capacity to enhance levels of endothelial nitric oxide synthase (eNOS) and nitric oxide [58] in a rat model exhibiting glucose-induced endothelial dysfunction. The administration of ASIV at doses of 40 or 80 mg/kg body weight per day to Sprague Dawley rats resulted in pronounced anti-inflammatory and antioxidant effects, which may elucidate the protective properties of this triterpenoid saponin on vascular endothelium.

Zhang and co-workers have listed other signaling pathways that may be affected by astragaloside IV administration [59]. In a mouse model of endometriosis, ASIV was shown to suppress endometriosis-induced inflammatory lesions and attenuate TLR4/NF-ĸBsignaling activated in vivo in endometriosis. Along with this effect, the reduced expression of TNF-alpha, Ccl-2, IL-1beta, and IL-6 was observed, resulting in a reduced inflammatory state. The influence on the TLR4/NF-ĸB signaling pathway was also listed by other authors in a high glucose-induced NLRP3 inflammasome model [60]. In addition to the aforementioned mechanisms, these authors also emphasized the ability of ASIV to decrease the levels of IL-18, NLRP3, caspase-1, ASC, nuclear p65, CaSR, and TLR4. These additional capabilities demonstrate its broad effects in a living organism and provide further insight into its anti-inflammatory activity.

One target of ASIV discovered in the rat model of induced lung injury was reported by Wu et al. [61]. The administration of 50 or 100 mg/kg ASIV prior to the induction of injury resulted in a reduction in the consequences of lung injury. After treatment with this saponin, the dry-to-humid ratio of the lung was lowered, oxidative damage to the lung tissue was reduced, and the levels of IL-6, TNF-alpha, and CRP were lowered. The study highlighted the role of ASIV in modifying the TLR4/MyD88/NF-κBsignaling pathway by ASIV, providing further evidence of its anti-inflammatory effects.

Liu et al. [62] reported reduced IL-6 and TNF-alpha levels in mouse serum after ASIV treatment, using a psoriasis-like model to produce skin lesions and inflammation. The inhibition of the inflammatory state induced by ASIV was related to its interaction with the NF-ĸB signaling pathway.

Recent studies open up new possibilities for the use of ASIV in therapeutic strategies, i.e., by investigating the potential interactions with other drugs in search of potential synergistic effects.

In the study on the effects of astragaloside IV alone and together with tanshinone IIA from sage on myocardial ischemia-reperfusion injury [63], the authors demonstrated the ability of the combination to achieve better results and faster recovery. Both groups of animals treated with ASIV alone and the combination of Ta-IIA and ASIV showed lower CK, CKMB, and LDH levels, with the combination showing more significant results. Reduced infarct area also characterized the treated groups, as well as improved muscle contractility, especially when treated with a medium dose of ASIV together with Ta-IIA. Further studies on the predominant mechanisms of action emphasized the ability of ASIV to exert an anti-inflammatory and antioxidant effect by decreasing DHE fluorescence and MDA levels and increasing the activities of SOD and GSH. As for the anti-inflammatory effect, ASIV was able to decrease the mRNA expression of IL-6, IL-1beta, iNOS, and TNF-alpha in myocardial tissue. For these effects, the combination of two drugs was also more effective than a single administration of Ta-IIA or ASIV.

In summary, ASIV appears as a multifaceted anti-inflammatory agent with potential usage in various disease contexts and the treatment of different ailments that develop based on the inflammatory state. As outlined above, the anti-inflammatory capabilities of ASIV can have various applications in clinical pharmacology as the substance can target different inflammatory mechanisms, including modulating the PPAR-alpha/FSP1, JAK/STAT, TLR4/Myd88/NF-κB, and attenuating the TLR4/NF-κB signaling pathways.

Its ability to decrease the levels of IL-1β, IL-6, IL-18, TNF-α, CCL-2, GSDMD-N, NLRP3, cleaved caspase-1, caspase-1, ASC, nuclear p65, CaSR, and TLR4, and the expression of JAK2, STAT1, STAT3, VCAM-1, ICAM-1was proved.

Fortunately, the number of scientific publications on the anti-inflammatory potential of ASIV demonstrated in in vivo studies has increased significantly in recent years. Certainly, the studies on the potential of ASIV do not yet fully explain the influence of this molecule on the animal organism. However, they provide clues for future promising applications of this triterpenoid saponin in human studies. Certainly, further tests on an effective formulation for ASIV should be developed to allow the efficient utilization of its pharmacological potential. For the time being, the polyvinyl alcohol-based nanofibers containing polysaccharides from Astragalus, together with astragaloside IV-loaded liposomes, exerted anti-inflammatory effects when applied topically in a wound inflammation model in diabetic rats [64]. The topical administration of the dressing material resulted in a clear regeneration of diabetic wounds through its ability to regenerate the epithelium, improve collagen fiber synthesis, and strengthen the overall wound healing process. During treatment, a reduction in wound area was observed, along with improved tissue proliferation and cell adhesion, which promoted faster tissue regeneration. Within the 15-day treatment, neutrophil counts decreased and the overall inflammatory state was alleviated more rapidly in the treated groups compared to the control group, suggesting the regenerative properties of astragaloside IV and polysaccharides from *Astragalus*. Selected in vivo models used to investigate the anti-inflammatory properties of ASIV are presented in Table 5.

The aforementioned study serves as an important example of the application of ASIV, highlighting the utilization of its biological properties and presenting further pharmacological effects of ASIV when administered in an efficient pharmaceutical form. It is anticipated that future studies will provide additional examples of its application, potentially enabling the incorporation of ASIV into the treatment of inflammation-based diseases.

## 5. Neuroprotection and Cognition Enhancement

The anti-inflammatory potential of ASIV has been demonstrated to be beneficial in the assessment of its impact on neurological disorders (see Figure 3). Civilization diseases related to memory impairment and other central nervous system dysfunctions are a growing problem these days. In addition to the reported disturbances in the levels of neuromodulators or the harmful effects of reactive oxygen species, these diseases progress with the development of inflammation in the CNS. For this reason, the anti-inflammatory properties of a drug may significantly attenuate the consequences of these slowly progressive diseases [65] and promise a better prognosis for patients treated with standard drugs.

Encouragingly, astragaloside IV alone and as the major component of Astragalus extract has been identified as possessing anti-inflammatory, neuroprotective, and cognition-enhancing properties. Numerous studies are currently investigating its application in the treatment of neurological disorders, including Alzheimer’s disease, various forms of memory impairment, Parkinson’s disease, neurological tumors, stroke, depression, and cerebral ischemia [66,67].

A large share of scientific publications describe the role of ASIV in reverting the symptoms of subcortical ischemic vascular dementia (SIVD) and vascular dementia (VaD) [68].

The latter, defined as a syndrome of severe cognitive impairment, is the most common cause of dementia and other neurological disorders in the elderly.

The clinical manifestation of this disorder is cognitive dysfunction, mainly caused by white matter hypoperfusion due to the constriction of small blood vessels [68,69,70]. From a molecular point of view, sirtuins or SIR proteins play a key role in the regulation of the inflammation, neuronal metabolism, and neurodegeneration observed in this condition [68,71,72,73]. The activation of the SIRT1 protein and a resulting significant reduction in oxidative stress triggered by the administration of ASIV was largely responsible for the mildening of cognitive dysfunction [68,74,75]. Treatment with ASIV diminished the activation of glial cells, promoted the formation of oligodendrocytes, and increased expression levels of HO-1, Nrf2, and NQO1 [68].

This study underscores the role of ASIV in the regulation of nuclear factor erythroid 2 (Nrf2) expression. This transcription factor is believed to maintain redox homeostasis and promote the transcription of enzymes that counteract the oxidation of genes [76]. An increase in Nrf2 activity leads to reduced effects of oxidative stress, which can contribute to stroke, thereby decreasing the likelihood of brain damage [77]. The activation of Nrf2 is regulated by SIRT1, and the SIRT1/Nrf2 pathway has been shown to exert protective effects on neurons [65,78,79,80,81].

Another potential mechanism of ASIV action was demonstrated by Liu et al., who found that intraperitoneal injection of Astragalus extract inhibited neuronal apoptosis and the expression of the apoptosis-related gene JNK3 in the context of cerebral ischemic injury [82]. Consequently, treatment resulted in a reduction ininfarct volume, as well as improvements in the shape, structure, and function of neurons. In a separate study, the intravenous administration of ASIV was shown to prevent the infiltration of leukocytes into ischemia-reperfusion tissue of the brain, thereby mitigating inflammation and brain swelling [66,67,83].

Among the various protective properties of ASIV, Liu et al. [84] also demonstrated its ability to inhibit X-ray-induced neuronal damage through the activation of the BDNF-TrkB signaling pathway. This effect was observed following the administration of ASIV to mice at a dose of 40 mg/kg body weight per day via intraperitoneal injection for four weeks prior to X-ray irradiation. The observed effects of this saponin were associated with the restoration of homeostasis, including the upregulation of neuron-related genes, increased levels of F-actin and PSD-95, the correction of memory deficits, enhanced specialized learning, and an increased dendritic complexity index.

Further properties of ASIV were described in the study by Chen et al. [85] in rats. The authors confirmed the previous results of the in vitro studies, indicating the ability of ASIV to induce brain repair via EGF-MAPK signaling, in tests on post-ischemic rat brains injected with 2 µg/kg ASIV i.v. for 7 days. The results of this study clearly indicate the ability of this substance to promote both astrogen formation and neurogenesis in different brain zones such as the cortex, dentate gyrus, or subventricular zone without affecting neuronal or astrocytic differentiation. In addition, infarct volume was reduced in rats, the proliferation of NSCs was promoted, and there was an increased rate of BrdU/SOX-2-, BrdU/DCX-, and BrdU/NeuN-positive staining cells.

Other authors also recognized various points of the complex mechanism of action of ASIV suitable for the treatment of injury-induced recovery. Wang et al. [86] proposed the P62/Keap1/Nrf2 signaling pathway as the target of ASIV in the cerebral ischemia-reperfusion injury model, which attenuated the cell damage and sensorimotor dysfunction induced by OGD/R in rats, increased the levels of Nrf2 and p62 proteins, and decreased Keap1 levels. Rao et al. [87] demonstrated the analgesic properties of ASIV in the spinal cord injury model, which may be an effect of interaction with the OIP5-AS1/miR-34a/Sirt1/NF-κB axis. Indeed, saponin alleviated neuropathic pain and miR-34a expression and increased the level of Sirt1 protein and OIP5-AS1 expression.

In addition to its effects on the state of neurons and astrocytes, ASIV has been shown to modify the secretion of neuromodulators. These properties were demonstrated in a post-stroke depression model, where an increase in dopamine and serotonin levels was observed in the brains of treated rats, along with an upregulation of the NRG-1-mediated MEK/ERK signaling pathway, contributing to more effective treatment of depression.

ASIV administered intraperitoneally to mice at a dose of 25 mg/kg attenuated scopolamine-induced memory impairment, suggesting a mechanism of action involving muscarinic cholinergic receptors. ASIV influenced both the consolidation of long-term memory and memory acquisition in the scopolamine group; however, no effect was noted in the model of LPS-induced memory impairment. Additionally, the impact of this compound on the synthesis of phosphatidylcholines was highlighted, indicating its potential as a memory-enhancing agent with pro-neurogenic properties.

Behavioral studies using the novel object recognition test and the Morris water maze test in a model of microglial activation and inflammation induced by intracerebroventricular injection of oligomeric amyloid-beta (oAβ) further underscored the potential application of ASIV in the treatment of dementia. Under the influence of this saponin, the cognitive impairment in animals was alleviated, microglial activation was inhibited, and the expression of NADPH oxidase proteins and neuronal damage were reduced. Furthermore, a decrease in interleukins IL-1β and IL-6, TNF-α, and reactive oxygen species (ROS) in the hippocampus was observed.

The underlying mechanism of the beneficial effects of ASIV in the treatment of Alzheimer’s disease has also been elucidated by Chen et al. [88]. In their study, ASIV was shown to suppress the reduction inbrain-derived neurotrophic factor (BDNF) induced by oligomeric amyloid-beta (AβO) in an experimental model by promoting peroxisome proliferator-activated receptor gamma (PPARγ) activation directly in the hippocampus. Additionally, Xia et al. [89] suggested that ASIV may enhance the longevity of astrocytes and protect them from aging. Immunohistochemical analyses revealed a reduced loss of dopaminergic neurons in a mouse model of Parkinson’s disease. The study documented a decreased number of senescent astrocytes in the substantia nigra compacta region of the brain, accompanied by reduced reactive oxygen species (ROS) generation and a lower number of damaged mitochondria in the brain tissue.

Aqueous extracts containing this saponin have demonstrated the ability to improve cognitive functions impaired by Aβ25–35 amyloid plaques in mice [90]. The extract prevented the destruction of synapses and axons in the cerebral cortex and hippocampus while promoting the elongation of axon terminals in cortical neurons and facilitating the formation of synaptic gaps affected by the aforementioned plaques.

Moreover, a significant effect of ASIV on the regeneration of dopaminergic neurons was observed by Yang and colleagues in a similar model of Parkinson’s disease. In addition to the previously mentioned properties, the inhibition of inflammation and oxidative stress in brain tissue also contributed to the restoration of the physiological state of the animals.

The number of studies investigating the effects of ASIV on CNS functions is substantial and continues to grow each year. This phenomenon reflects a burgeoning interest in the therapeutic potential of ASIV, particularly in the context of neurological disorders. Initial studies conducted in vitro have laid the groundwork for understanding the mechanisms through which ASIV exerts its effects. These foundational experiments have provided valuable insights into the molecular pathways involved, including neuroprotection, the modulation of neurotransmitter systems, and anti-inflammatory actions.

As research has progressed, there has been a notable shift toward in vivo testing, which has resulted in promising results for the beneficial administration of ASIV in treating CNS disorders such as Alzheimer’s disease, Parkinson’s disease, and other neurodegenerative conditions.

In addition to its strong pharmacological potential, ASIV has demonstrated neutral toxicological effects on living organisms [91]. This characteristic is particularly important in the development of therapeutic agents, as it suggests a favorable safety profile, making ASIV a compelling candidate for broader clinical application. The combination of efficacy and safety positions ASIV as a promising therapeutic option in the realm of CNS disorders.

Recent trends in the field of drug development, particularly the exploration of new dosage forms for natural products, could significantly enhance the therapeutic utility of ASIV. Innovative drug delivery systems aim to optimize the bioavailability and efficacy of compounds like ASIV, ensuring that they reach their target sites in the brain effectively. Such advancements could lead to the development of efficient methods to maximize the effects of this saponin on brain tissue, improving treatment outcomes for patients with neurological disorders.

A particularly intriguing in vivo study conducted by Zhao et al. [92] has contributed to this area of research by developing a novel nose-to-brain delivery system for ASIV. This approach utilizes chitosan nanoparticles modified with beta-asarone to facilitate the delivery of ASIV directly to the brain. The nose-to-brain route is advantageous because it bypasses the blood–brain barrier, a significant obstacle in CNS drug delivery. By improving the penetration of ASIV into the brain, this innovative formulation may enhance its therapeutic effects and broaden its applicability in treating CNS disorders. Chosen in vivo models used to investigate the neuroprotective potential of ASIV are presented in Table 6.

Overall, the expanding research landscape surrounding ASIV highlights its potential as a valuable therapeutic agent for CNS disorders. Ongoing studies are likely to yield further insights into its mechanisms of action, safety, and efficacy. As new delivery methods are explored and developed, the hope is to translate these findings into effective clinical therapies that can significantly improve the lives of individuals affected by neurological conditions.

## 6. Anticarcinogenicity

Numerous experimental in vivo studies confirm the anti-cancer effect of astragaloside IV (see Table 7).

ASIV has also been shown to inhibit the progression of gastric cancer (GC) by targeting circDLST, which modulates the miR-489-3p/EIF4A1 signaling pathway. ASIV exhibited significant antitumor properties in the context of gastric cancer, affecting disease progression through its interaction with RNA molecules. The overexpression of circDLST was found to significantly counteract the inhibitory effect of ASIV on GC cells, indicating that ASIV suppresses the development of GC by downregulating circDLST. Additionally, prediction and targeting analyses identified miR-489-3p as a microRNA target associated with circDLST, suggesting that circDLST could potentially serve as a biomarker in ASIV-mediated gastric cancer therapy [94].

Moreover, ASIV was found to inhibit tumor growth and prolong the survival of tumor-bearing mice in vivo. Specifically, ASIV reduced the number of lung metastatic nodules compared to the control group. Furthermore, the expression of both CD-31 and VEGFA was decreased in tumor tissue after treatment with ASIV [95].

It has also been demonstrated that treatment with both ASIV and saponins can reverse the cyclophosphamide (CTX)-induced reduction in white blood cells (WBCs) and bone marrow hematopoietic stem cells (BMHSCs) in vivo. It is hypothesized that ASIV and saponins (SRP) may improve myelosuppression and enhance hematopoietic function, thus serving as potential therapeutic agents during chemotherapy. Both ASIV and SRP inhibited BMHSC damage by triggering an increase in Bcl-2 levels and a decrease in Bax and cleaved caspase-3 levels. This change in BMHSC damage following CTX treatment was attributed to the downregulation of miR-142-3p. Given that miRNAs play a critical role in regulating numerous target genes, it was demonstrated that ASIV and SRP can attenuate CTX-induced injury to BMHSCs through this mechanism [96].

Another study explored the impact of ASIV for suppressing cervical cancer cell invasion when dosed at doses of 12.5, 25, and 50 mg/kg b.w.in xenograft mice. The 35-day-long treatment led to the shrinking of the cancer tissue starting from the middle dose [97].

Further research revealed that ASIV effectively suppressed the growth of uterine leiomyomas (ULMs) in vivo and exhibited potent antitumor activity. ASIV promoted autophagy in ULM rats by upregulating levels of LC3 and FOXO3A. Western blot and immunohistochemistry results indicated that ASIV significantly reduced the protein expression of IDO1, PI3KCA, and AKT1 while increasing PTEN protein expression. Based on the findings of an in vivo study, it is proposed that ASIV promotes apoptosis and autophagy in ULMs by modulating the PTEN/PI3K/AKT signaling pathway, with IDO1 playing a critical role in this mechanism [98].

In an in vivo model, mice with CT26 tumors were treated with three doses of ASIV (15.0 mg/kg) every three days, resulting in reduced tumor growth, as evidenced by decreased tumor volume and weight compared to PBS-treated controls. Flow cytometric analysis showed that ASIV treatment decreased the proportion of CD11b^+^ F4/80^+^ CD206^+^ M2 macrophages, while the proportion of CD11b^+^ F4/80^+^ MHCII^+^ M1 macrophages increased in tumor tissue, further confirming the shift from M2 to M1 polarization in vivo. ASIV was also shown to significantly inhibit M2 polarization in vivo and influence tumor development. Chronic inflammation is recognized to contribute to carcinogenesis, with macrophage polarization being a critical factor in the inflammatory microenvironment. Treatment with ASIV reduced anti-inflammatory cytokines such as TGF-β, IL-10, and VEGF-A, while pro-inflammatory cytokines such as IFN-γ, IL-12, and TNF-α were increased in tumor tissue. ASIV is thought to modulate the inflammatory microenvironment and M2 macrophage polarization. Combination therapy with ASIV and immune checkpoint inhibitors proved to be even more effective as it suppressed tumor growth and enhanced T-cell infiltration more effectively than ASIV alone. In addition, the combination treatment lowered levels of immunosuppressive cytokines and significantly increased immune-activating cytokines such as IL12p70 and IFN-γ. These results suggest that ASIV not only regulates macrophage polarization but can also act synergistically with immune checkpoint inhibitors, which is a promising therapeutic strategy for colorectal cancer (CRC) [99].

ASIV also actively diminished the growth of liver cancer in the BALB/c nude mice xenograft model with Huh-7 cells when administered once daily for 40 consecutive days. The tumor growth decreased proportionally to the increasing dose of ASIV and to the simultaneous reduction inPD-L1 expression indicating the immunomodulatory mechanism of the saponin [100].

In conclusion, the extensive number of in vivo studies on ASIV supports its anti-cancer activity potential. The modulation of macrophage polarization, particularly the shift from pro-inflammatory M1 to pro-resolving M2 phenotypes, appears to be a critical mechanism underlying ASIV’s therapeutic effects. ASIV’s ability to modulate the key transcription factors, such as STAT1 and STAT3, indicates its potential to restore immune balance and promote tissue homeostasis. ASIV was found to be efficient in inhibiting gastric cancer progression and has been shown to prolong survival in tumor-bearing models while reducing metastatic burden, further establishing its role as a potent anti-tumor agent. The compound also exhibits protective effects against chemotherapy-induced myelosuppression, enhancing hematopoietic functions and thus reducing damage to bone marrow stem cells, which is of particular importance in patients directed to chemotherapy. It is worth noting that ASIV induces apoptosis in various cancer cell types through mitochondrial pathways and caspase activation, reinforcing its potential as a therapeutic agent in colorectal cancer and other malignancies. Importantly, ASIV’s role in modulating the tumor micro environment, particularly regarding macrophage polarization and immune regulation, is also promising in combination therapies with immune inhibitors. Such synergistic approaches may enhance anti-tumor immune responses and improve treatment outcomes in colorectal and hepatocellular cancers. Certainly, this direction of study is of particular importance. The findings also suggest that ASIV influences metabolic pathways in cancer cells, potentially slowing down the tumor progression, e.g., through the regulation of succinylation and glycolysis. The identification of molecular targets such as PGAM1 highlights the complexity of ASIV’s mechanisms of action and its potential for broader therapeutic applications. Overall, the gathered evidence positions ASIV as a multifaceted compound with significant promise for the treatment of various cancer types that should be further studied to elucidate its mechanisms and pave the way toward its clinical application.

A comparative figure showing selected mechanisms of anti-cancer, anti-inflammatory, and neuroprotective effects of ASIV is shown in Figure 3.

**Table 7:** In vivo models to study the anticancer effect of astragaloside IV.

Type	Organism	Dose	Mechanism	References
Sanhuang decoction	Wistar male rats weighing 150–160 g, clean grade	0.5 g/kg SASP enema (10 mL/kg b.w.) and 60 g/kg MSD enema (10 mL/kg b.w.)	The TNF-α, IL-1β and IL-6 levels in the MSD group significantly decreased	[101]
Sanhuang decoction	Female nude mice (6 weeks old, 18–22 g)	4 g/kg	Positive role in inhibit ing the growth of MCF-7 cancer xenografts in vivo, the expression of IL-6 and TNF-α was observed to be obviously de- creased, the expression of VEGF, MMP-2, and MMP-9 was observed to be down-regulated in the Sanhuang decoction treatment group	[102]
ASIV	Male C57BL/6J mice (5 weeks old)	40 mg/kg, 80 nM	Ameliorated cancer-associated inflammation, de- creased the expression of inflammatory factors such as TGF-β and IL-10, and suppressed M2 macro- phage polarization and de- creased the density of M2 macrophages	[95]
ASIV with saponins and cyclophosphamide	Male BALB/c nude mice (6weeks old, weighing 20 ± 2 g)	20 mg/kg/day, 5 μM	AS and SRP improved the hematopoietic function and cured myelosuppression in CTX- induced myelosuppression mice	[96]
ASIV	Four-week-old BALB/c nude mice weighing 15–17 g	25 mg/kg/d, 25 μM	Decreased growth of cervical cancer tumor	[97]

## 7. Future Perspectives

Growing evidence regarding a multitude of biological propertiesand the beneficial safety profile of astragaloside IV—themostactive constituent of *Astragalus membranaceus*–promises future application of the saponin in conventional medicine. As discussed in the review, ASIV showed significant efficiency in a multitude of preclinical models conducted toward the determination of its anti-inflammatory, neuroprotective, cognition-enhancing, anticancer, and antioxidant properties, which suggests the possibility of its use in the treatment of various civilization diseases that modern societies are struggling with.

To achieve this goal, the transition from preclinical findings to clinical applications is crucial. Future studies should focus on designing and conducting well-organized clinical trials to evaluate the safety, efficacy, and optimal dosing regimens of ASIV in human populations suffering from chronic inflammatory diseases, neurodegenerative disorders, and various cancers. These studies should also explore the potential synergistic effects of ASIV when combined with conventional therapies, such as chemotherapy and immunotherapy.

Clinical studies on humans could open the doors for further discussions on the molecular mechanisms of action and specific signaling pathways influenced by ASIV, particularly in relation to macrophage polarization and immune modulation. Identifying key molecular targets and understanding the interactions between them could provide deeper insights into ASIV’s activity and, through this information, may facilitate the development of more targeted therapies. As ASIV progresses toward clinical application, comprehensive safety evaluations and regulatory compliance will be paramount. Future research should include rigorous toxicity studies and long-term safety assessments to ensure patient safety and facilitate regulatory approval. As proven by some initial studies, the pharmacological potential of ASIV may be fueled by the introduction of new drug delivery forms that could optimize and facilitate a better pharmacokinetic profile of this metabolite. Research into advanced formulations, such as nanoparticles, liposomes, and sustained-release systems, could enhance the bioavailability and targeted delivery of ASIV to specific tissues, particularly in the central nervous system (CNS). The implementation of new delivery methods, such as the nose-to-brain route, may significantly improve therapeutic outcomes for neurological conditions. The potential for ASIV to act synergistically with other therapeutic agents, particularly in the context of immune modulation and cancer therapy, should be a focus of future studies as well. Investigating combination therapies that incorporate ASIV alongside immune modulators or other novel agents may enhance treatment efficacy and improve patient outcomes, as was the case of the previously discussed ASIV and gefitinib or ASIV and cisplatin connections. In conclusion, the extensive research surrounding ASIV underlines its potential as a multi-target therapeutic agent that can be applicable in various inflammatory, cognitive, and oncological conditions. By addressing the outlined perspectives, researchers can open the door for the introduction of ASIV into clinical practice and improve the treatment options and outcomes for patients affected by chronic civilization diseases. Continued exploration of ASIV will not only reveal its mechanisms of action but also expand its therapeutic horizons, contributing to the advancement of medicine and improved therapeutic strategies.

## Figures and Tables

**Table 1:** Physicochemical properties of astragaloside IV: TPSA, topological polar surface area; logP_ow_, logarithm of the *n*-octanol/water partition coefficient; logP_cw_, logarithm of the cyclohexane/water partition coefficient; logP_hw_, logarithm of heptane/water partition coefficient; ΔlogP, difference between the *n*-octanol/water and cyclohexane/water logP values [24,25].

Name of Property	Description/Value
Molecular weight	784.87 g/mol
TPSA	228.22 Å^2^
LogP_ow_	3.757
LogP_cw_	−9.015
LogP_hw_	−9.3
Physical Description	A crystalline solid
Color	White (yellow) crystallinepowder
Flash Point	495.5 °C
Melting Point	295–296 °C
Boiling Point	895.7 °C
Density	1.39 g/cm^3^
Solubility	DMF: 20 mg/mL DMSO: 30 mg/mL DMSO:PBS (pH = 7.2) (1:1 *v*/*v*): 0.5 mg/mL

**Table 2:** Mean values of the concentrations with standard deviations of ASIV in selected organs of rats at an intravenous dose of 4 mg/kg for *n* = 6 [19,28].

Organ	Mean Concentration [ng/mL]	Standard Deviation [ng/mL]
Liver	916	506
Kidneys	587	301
Lungs	463	494
Spleen	216	114
Heart	90.9	45.7

**Table 3:** Values for the elimination time (T_1/2_), the area under the ASIV concentration–time curve (AUC), and the mean maximum concentration of astragaloside in human plasma (C_max_) determined in studies in healthy patients [19,31].

Dose [mL]	T_1/2_ [h]	AUC [μgh/mL]	C_max_ [g/mL]
200	2.14	4.38	2.12
300	2.59	9.75	3.59
400	2.62	13.59	3.71
500	2.69	18.22	5.17

**Table 4:** Values of hepatic clearance (C_l_), hepatic blood flow (F_l_), area under the curve of ASIV concentration vs. time (AUC), mean maximum concentration of ASIV in human plasma (C_max_), and absolute bioavailability (EBA) determined in studies in dogs and rats [29,32].

Animals	C_l_ [mg/kg/min]	F_l_ [%]	AUC [mgh/mL]	C_max_ [mg/mL]	EBA [%]
Rats	3.00	5.43	289.16	3.78	2.20
Dogs	4.00	12.90	156.04	4.39	7.40

**Table 5:** In vivo models to study the anti-inflammatory potential of astragaloside IV(↓—decrease/alleviation, ↑—increase/promotion).

Drug	Model	Animals	Dose	Effect	References
ASIV	Diabetic kidney disease	6-week-old rats	10 or 20 mg/kg b.w./day for 8 weeks	↓ blood glucose level ↓ ratio of urinary albumin to creatinine ↓ disorder of lipid metabolism ↓ injury in diabetic kidneys ↓ proteinuria	[53]
ASIV	Acute kidney injury	4-week-old SpragueDawley (SD) male rats	5 or 10 mg/kg b.w.p.o. ASIV	no inflammatory infiltration ↓ necrosis of epithelial cells ↓ BUN, SCR levels ↓ IL-18, IL-1beta, GSDMD-N and cleaved-caspase-1 levels	[54]
ASIV	Cisplatin-induced liver injury	Mice	40 or 80 mg/kg b.w.	Significant improvement in inflammatory and oxidative stress conditions; inhibition of ferroptosis	[55]
ASIV	Psoriasis model of skin lesions and inflammation			↓ IL-6 ↓ TNF-alpha	[62]
Glucosides (ASIV, paeoniflorin, amygdalin) and *BuyangHuanwu Decoction*	Atherosclerotic inflammation	6–8-week-old ApoE−/− and C57BL/6J mice	4 weeks, 2.772 g/kg/day of BYHWD, 0.167 or 0.084 g/kg/day of glucosides	↓ inflammatory response ↓ protein expression of JAK2, STAT1, STAT3, VCAM-1, ICMA-1proteins, IL-6, and TNF-alpha in aorta wall ↓ TC, TG, LCLC-c ↑ HDL	[57]
ASIV	Lung injury model	Male SpragueDawley rats	50 or 100 mg/kg b.w. ASIV	↓ lung injury ↓ lung dry–wet ratio ↓ IL-6, TNF-alpha, CRP ↓ oxidative response in lung tissue Impact on TLR4/MyD88/NF-κB pathway	[61]
ASIV and Astragalus polysaccharides-loaded nanofibers	Diabetic rat wound inflammation model	Four groups of rats weighing 180–220 g	PVA nanofibers with ASIV and polysaccharides from *Astragalus* for 15 days	↓ wound area ↑ tissue proliferation No infection ↑ cell adhesion ↑ cell migration ↓ neutrophils ↓ inflammation ↑ collagen fibers	[64]
ASIV	High glucose-induced endothelial dysfunction model	Sprague Dawley rats	40 or 80 mg/kg/day of ASIV for 8 weeks	↑ endothelial relaxation ↑ eNOS ↑ NO ↓ inflammation and oxidative stress in diabetic model	[58]
ASIV	Endometriosis	6-week-old female mice	0,5, 10 or 30 mg/kg b.w./day for 5 weeks	↓ inflammation ↓ TLR4/NF-ĸBsignaling ↓ expression ofTNF-alpha, Ccl-2, IL-1beta and IL-6	[59]
ASIV with tanshinone IIA	Myocardial ischemia (30 min) and infarction	8–9-week-old male C57BL/6 mice	i.p. injections of 15 mg/kg/day ASIV, 10 mg/kg/day Ta-IIA, or in combination: ASIV (15 mg/kg) + Ta-IIA (10 mg/kg) or ASIV (10 mg/kg) + Ta-IIA (5 mg/kg) or Ta-IIA (15 mg/kg) + ASIV (20 mg/kg)	↓ mRNA expression of IL-6, IL-1beta, iNOS, TNF-alpha ↑ SOD and GSH levels	[63]

**Table 6:** In vivo models to study the neuroprotective properties of astragaloside IV(↓—decrease/alleviation, ↑—increase/promotion).

Drug	Model	Animals	Dose	Effect	References
ASIV	Radiation-induced neuronal damage	Mice Thy1-YFP line H	40 mg/kg b.w./day ASIV, i.p. for 4 weeks	↑ BDNF-TrkB signaling ↑ (*Ngf*, *Bdnf*, *Gap-43*, *Ras*, *Psd-95*, *Arc*, *Creb*, *c-Fos*) genes, PSD-95 and F-actin	[84]
	Memory impairment model with scopolamine	Swiss-type mice	25 mg/kg b.w.i.p. ASIV	↑ memory impairments ↑ phosphatidylcholine level	[91]
	Cerebral ischemic injury model	SpragueDawley rats 260–280 g	2 µg/kg/day ASIV i.v. for 7 days	↑ EGRF/MAPK cascades ↑ astrogenic and neuronal formation	[85]
	Post-stroke depression model	Male Sprague Dawley rats 200–240 g	2 μg/kg ASIV i.v. for 7 days	↑ NRG-1-Mediated MEK/ERK Pathway ↑ dopamine ↑ serotonin	[93]
	Cerebral-ischaemia reperfusion injury model	Male Sprague Dawley rats 5–7 week-old 200–220 g	28 mg/kg ASIV i.g.	↑ P62/Keap1/Nrf2 pathway ↓ ferroptosis, cerebral reperfusion injury ↓ MCAO/R-induced brain damage ↑ Nrf2 protein, p62 ↓Keap1	[86]
	Spinal cord injury model	Sprague Dawley rats 5–8 weeks old, 18–220 g	20 mg/kg b.w./day, i.p.	↑ OIP5-AS1 ↑ Sirt1 ↓ neuropathic pain ↓ miR-34a	[87]
	Oligomeric Aβ-induced Alzheimer’s Disease mouse model	Male ICR mice 22–25 g	20, 40, 80 mg/kg b.w./day i.g. ASIV	↓ IL-1beta, IL-6, TNF-alpha, ROS ↓ microglial activation, ↓ NADPHoxidase protein expression ↓ neuronal damage	[88]
	Model of Parkinson’s disease	Mice	20 mg/kg ASIV	↓ astrocytes senescence ↑ dopamine neurons ↓ ROS, damaged mitochondria in substantia nigra	[89]

## Data Availability

Not applicable.
